# Prevalence of iodine deficiency and associated factors among pregnant women in Ada district, Oromia region, Ethiopia: a cross- sectional study

**DOI:** 10.1186/s12884-018-1905-z

**Published:** 2018-06-25

**Authors:** Mengistu Fereja, Samson Gebremedhin, Tafere Gebreegziabher, Meron Girma, Barbara J. Stoecker

**Affiliations:** 1 School of Nutrition, Food Science and Technology, Hawassa University, P.O.Box 05, Hawassa, Ethiopia; 2 School of Public and Environmental Health, Hawassa University, Hawassa, Ethiopia; 3 Department of Nutritional Sciences, Oklahoma State University, Stillwater, OK USA

**Keywords:** Pregnant women, Urinary iodine, Goiter, Iodine deficiency, Iodized salt

## Abstract

**Background:**

Maternal iodine deficiency (ID) during pregnancy has been recognized as a major cause of abortion, stillbirth, congenital abnormalities, perinatal mortality and irreversible mental retardation. In Ethiopia limited information is available regarding the epidemiology of maternal ID. The purpose of the present study was to assess the prevalence of iodine deficiency and associated factors among pregnant women in Ada district, Oromia region, Ethiopia.

**Method:**

A community based, cross-sectional study was conducted in rural areas of Ada district, October to November, 2014. Data were collected from 356 pregnant women selected by multistage cluster sampling technique. Presence of goiter was examined by palpation and urinary iodine concentration was measured using inductively-coupled-plasma mass spectrometry. Salt iodine concentration was determined using a digital electronic iodine checker. Statistical analysis was done primarily using binary logistic regression. The outputs of the analysis are presented using adjusted odds ratio (AOR) with the respective 95% confidence intervals (CI).

**Results:**

The median urinary iodine concentration (UIC) was 85.7 (interquartile range (IQR): 45.7–136) μg/L. Based on UIC, 77.6% (95% CI: 73.0–82.0%) of the study subjects had insufficient iodine intake (UIC < 150 μg/L). The goiter rate was 20.2% (95% CI: 16.0–24.0%). The median iodine concentration of the household salt samples was 12.2 (IQR: 6.9–23.8) ppm. Of the households, only 39.3% (95% CI: 34.0–44.0%) consumed adequately iodized salt (≥15 ppm). Prevalence of goiter was significantly higher among pregnant women aged 30–44 years (AOR = 2.32 (95% CI: 1.05–5.14)) than among younger women and among illiterate women (AOR = 2.71 (95% CI: 1.54–4.79)). Compared to nulliparous, women with parity of 1, 2 and 3 or more had 2.28 (95% CI: 1.01–5.16), 2.81 (95% CI: 1.17–6.74) and 4.41 (95% CI: 1.58–12.26) times higher risk of goiter.

**Conclusion:**

Iodine deficiency was a public health problem in the study area. This indicates the need for further strengthening of the existing salt iodization program in order to avail homogenously and adequately iodized salt. Also it is necessary to find ways to provide iodine supplements as needed until universal salt iodization (USI) is fully established.

## Background

Iodine is an essential micronutrient and component of the thyroid hormones which play key roles in normal growth, development and metabolism [1]. Insufficient iodine intake results in inadequate production of thyroid hormones which subsequently exerts effects on different organs and body systems and results in multiple adverse health effects collectively named as “iodine deficiency disorders (IDD)” [2].

Iodine deficiency is a global public health problem and affects both developed and developing countries [3]. In 2011, an estimated 1.8 billion people (28.5%) worldwide consume inadequate amounts of iodine and are at risk for IDD [4]. Pregnant women and their fetuses are particularly vulnerable to the wide spectrum of disorders caused by iodine deficiency. Consequences of iodine deficiency during pregnancy include increased risk of spontaneous abortion, stillbirth, retarded development of the fetus, low birth weight, perinatal death, and stunted infant growth, as well as neuromotor, intellectual, behavioral and cognitive impairments and neonatal hypothyroidism and goiter [5–8]; WHO states that “iodine deficiency is the world’s most prevalent, yet easily preventable, cause of brain damage” [2]. Despite this, currently there is inadequate data to estimate the prevalence of iodine deficiency during pregnancy in nearly all countries of the world [9].

Studies over the past decade have indicated the public health significance of IDD in Ethiopia. A national micronutrient survey in 2005 reported 35.8% total goiter rates (TGR) in women of reproductive age [10]. In 2011 the International Council for the Control of Iodine Deficiency Disorders (ICCIDD) estimated that 12 million school age children (SAC) were living with inadequate iodine (4) and around 66 million of the Ethiopian population were at risk of iodine deficiency [11]. A few studies before Ethiopian mandatory salt iodization program also reported a high level of iodine deficiency in pregnant women. A study conducted in Sidama zone reported a median urinary iodine concentration (UIC) of 15 μg/L and TGR of 49% [12]. Furthermore, a study conducted in 2012, a few months after mandatory salt iodization, reported a median UIC of 58 μg/L and UIC less than 150 μg/L in 82.8% of pregnant women [13]. Recognizing the public health significance of iodine deficiency in Ethiopia, the Council of Ministers passed new salt legislation in February 2011 and the government started implementing a mandatory salt iodization program and public promotion in 2012 [11]. However, studies addressing iodine status of pregnant women after the introduction of the mandatory salt iodization program are lacking in Ethiopia.

Thus, the purpose of the current study was to assess the prevalence of iodine deficiency and associated factors among pregnant women in rural Ada district, Oromia Region, Ethiopia, two years after the introduction of mandatory salt iodization.

## Methods

### Study design

This was a community based, cross-sectional, quantitative study with both descriptive and analytic elements.

### Study setting

The study was conducted in October and November 2014 in six rural kebeles of Ada district, Oromia region, Ethiopia. A kebele is the lowest administrative unit in Ethiopia containing approximately 1000 households. The area is located at 9°N latitude and 40°E longitude at an altitude of 1850 m above sea level (masl).

### Sample size

A single proportion sample size calculation formula was used to determine optimal sample size for estimating the prevalence of iodine deficiency. A sample size of 362 pregnant women was computed based on an estimated 82.8% prevalence of UIC < 150 μg/L [13], a 95% confidence level, 5% margin of error, design effect of 1.5 and non-response rate of 10%.

### Sampling technique

A multistage cluster sampling technique was used to select representative samples of pregnant women. Initially from the existing rural kebeles, six were selected based on the probability proportional to size sampling method. In every selected kebele, the households with pregnant women were identified through house to house visits by Community Health Promoters (CHPs) working at the sub-kebele level. The sampling frame was developed by registering all the identified eligible pregnant women in each kebele. Finally, individual subjects were selected using systematic random sampling technique.

### Data collection method

In the surveys, data were collected using a pre-tested structured questionnaire. Sociodemographic data were collected using questions adapted from Demographic and Health Surveys (DHS) questionnaire [14] and food consumption patterns were assessed using a standardized food frequency questionnaire [15]. The questionnaire was prepared in English and translated into the local language (Afan Oromo). The data were collected by three trained female nurse data collectors fluent in Afan Oromo.

Household economic status was assessed using composite indicators developed based on materials used for housing construction, ownership of selected household assets, size of agricultural land and quantity of livestock. Ultimately, a wealth index quantile was determined using Principal Component Analysis (PCA).

### Goiter examination

Presence of goiter was examined by one trained and experienced public health officer using palpation techniques and graded according to the criteria of WHO/UNICEF/ICCIDD [2]. Accordingly, grade 0 (no goiter), 1 (palpable but not visible goiter) and 2 (palpable and visible goiter.

### Salt and urine sample collection and laboratory analysis

Approximately 20 g of salt were collected from each pregnant woman’s home, packed in air tight plastic bags, and transported to the laboratory of the School of Nutrition, Food Science and Technology at Hawassa University for analysis. In addition a total of twenty six salt samples (50 g salt) were also collected/purchased from all the available shops in the selected kebeles. Salt iodine concentration was analyzed in duplicate using a WYD Iodine Checker (National Salt Research Center, Tianjin, China). The average of the two readings was taken as iodine concentration of the sample.

Casual urine samples were collected from each pregnant woman in disposable plastic cups and transferred into screw-capped plastic vials. Vials were sealed with Parafilm to prevent leakage and evaporation. The urine samples were frozen at − 20 °C until shipped to Oklahoma State University, USA for analysis. UIC was determined in duplicate using inductively coupled plasma-mass spectrometry (ICP-MS Elan 9000, Perkin Elmer, Norwalk, CT). The UIC was categorized according to the WHO/ICCIDD/UNICEF recommended epidemiological criteria for assessing iodine intake in pregnant women. Accordingly, UIC of < 150 μg/L, 150–249 μg/L, 250–499 μg/L, and ≥ 500 μg/L represent insufficient, adequate, more than adequate, and excessive levels of iodine intake respectively [2].

A total of six water samples were collected from publicly used borehole water and iodine concentration was analyzed by inductively-coupled plasma/mass spectrometer.

### Data analysis

The data were analyzed using SPSS v. 20. Histograms and the Kolmogorov-Smirnov test were used to check for normality of numeric data before further analysis. Descriptive analysis was done using mean, median (interquartile range (IQR)) and percentage as appropriate for categorical and continuous variables. UIC was skewed and not normally distributed. Non-parametric Mann-Whitney U test and Kruskall-Wallis test were used to compare the median UIC and salt iodine concentration and differences between groups of categorical independent variables.

Bivariate and multivariate logistic regression analyses were used to identify the variables associated with goiter status. Independent variables were selected and included in the analysis based on the review of existing literature about their suspected effects on the development of goiter. In multivariate logistic regression analyses, variables were entered separately into two models, distal and proximate. Factors which can have direct effect on goiter were considered proximate factors whereas factors which function through the proximate factors to affect goiter formation were considered distal factors. The distal model comprised maternal education, wealth index and awareness of iodized salt and IDD. The proximate model included maternal age, parity, gestational age, frequency of goitrogenic food consumption, salt iodine level, time salt was added during cooking and salt sun light exposure practice. The assumptions of logistic regression analysis (absence of multicollinearity) were checked. Goodness of fit of logistic models was assessed using Hosmer-Lemeshow statistic.

## Results

### Background information of study subjects

Of the 362 pregnant women recruited, data were successfully gathered from 356 women with a response rate of 98.3%. The median age of the respondents was 24 (IQR: 21–27) years. Gestational age at recruitment was first trimester for 32 (9.0%) women, second trimester for 94 (26.4%) and third trimester for 230 (64.6%). The median number of pregnancies was 2 (IQR: 1.0–3.0). Of the women one hundred sixty (45.0%) were illiterate and nearly all (95.2%) were not employed outside the home. The majority (83.4%) were Oromo in ethnicity and Orthodox Christians (96.3%). The average family size was 3.4 (±1.6) persons. The average size of agricultural land per household was 0.40 (±0.24) hectares (Table 1).

**Table 1:** Socio-demographic characteristics of study subjects in rural Ada districts, Oromia region, Ethiopia, November, 2014

Socio-demographic variable	Frequency (*n* = 356)	Percentage
Age (years)		
15–29	308	86.5
30–44	48	13.5
Educational status		
Illiterate	160	45.0
Informal education	8	2.2
Primary (1st- 4th grade)	36	10.1
Secondary (5-8th grade)	119	33.4
High school or above	33	9.3
Occupation		
Unemployed	339	95.2
Others	17	4.8
Ethnicity		
Oromo	297	83.4
Amhara	54	15.2
Others	5	1.4
Religion		
Orthodox	343	96.4
Protestant	8	2.2
Other	5	1.4
Marital status		
Married/Living together	352	98.9
Others	4	1.1

### Knowledge of iodized salt and iodine deficiency disorder and practice of use of iodized salt

Twenty-six percent of the respondents indicated that they had heard about iodized salt. For these women, the principal sources of information were health extension workers (HEW) (31.9%) and mass media (through radio) (30.9%). Only about one-fourth (21.6%) of the respondents had ever heard of goiter.

During the time of the survey 94.4% of the women used coarse salt (large crystals) and 5.4% used fine (powdered) salt. Concerning storage of salt at home, nearly all (98.9%) of the respondents stored the salt in a container with a lid. About 10% exposed salt to sunlight to dry it. Half of the respondents reported that they added salt towards the end of cooking and only 8.1% added salt after cooking.

### Iodine concentration of household salt

The iodine concentration (ppm) in the salt samples from the households ranged from 0.9 to 191.2 ppm with the median iodine concentration of 12.2 (IQR: 6.9–23.8) ppm. The median salt iodine concentration for fine and coarse salt type was 29.7 and 11.9 ppm, respectively. The difference was statistically significant (*P = 0.002*). Of the women 39.3% (95% CI: 34.0–44.0%) consumed adequately iodized salt (≥15 ppm) and 60.7% consumed salt with inadequate or almost negligible iodine content (Table 2). The median iodine concentration of the salt samples from retail shops was 15.0 (IQR: 9.4–46.8) ppm. The values ranged from 3.3 to 95.4 ppm.

**Table 2:** Household salt iodine concentration in six kebeles of rural Ada districts, Oromia region, Ethiopia, November, 2014 (*n* = 351)

Salt iodine (ppm)	Frequency	Percentage (%)
< 5	45	12.8
5–14.9	168	47.9
15–40	85	24.2
> 40	53	15.1

### Iodine concentration in drinking water

In each selected kebele, one sample of water was collected from the source most frequently used by the subjects. Median iodine concentration was 0.97 (IQR 0.01–7.4) μg/L.

### Urinary iodine concentration

The median UIC was 85.7 (IQR: 45.7–136) μg/L. The value ranged from 1.3 to 1147 μg/L. TheUIC of the women in the first trimester was 168.4 μg/L, 92.8 μg/L in the second and 74.1 μg/L in the third. The difference of the UIC in the three trimesters was statistically significant (*P = 0.001*). According to the criteria recommended by WHO/UNICEF/ICCIDD [2], 270 [77.6% (95% CI: 73.0–82.0%)] of the pregnant women had a UIC below 150 μg/L indicating insufficient iodine intake. Only 16.1% had adequate iodine level (150–249 μg/L) and 6.3% of the women had more than adequate iodine (above 249 μg/L) (Table 3).

**Table 3:** Urinary iodine concentration levels of pregnant women in rural Ada districts, Oromia region, November, 2014 (*n* = 348)

Urinary iodine concentration	Percentage (%)
Inadequate (< 150 μg/L)	77.6
Adequate (150–249 μg/L)	16.1
More than adequate (250–499 μg/L)	3.7
Excessive (> 500 μg/L)	2.6

### Prevalence of goiter

The prevalence of goiter among the pregnant women in the study area was 20.2% (95% CI: 16–24) and 79.8% had no goiter (Grade 0). Visible goiter (Grade 2) was not found in any of the study subjects. The prevalence of goiter during the first, second and third trimesters was 12.5, 18.1 and 22.2%, respectively.

### Factors associated with goiter status

Through bivariate and multivariate logistic regressions, the variables associated with goiter status were identified. Thirteen factors were considered as explanatory variables. In the bivariate analysis, maternal age, educational status and parity were significantly associated (*P < 0.05*) with the goiter status of women during pregnancy. However, household wealth index, gestational age, kale, cabbage and sorghum consumption, awareness about iodized salt and IDD, time salt added during cooking, salt sun light exposure practice and iodine level of salt were not significantly associated with goiter status.

In a multivariable analysis, the factors significantly associated with the goiter status of women in the bivariate analysis still remained significant. Accordingly, compared to literates, the illiterate women had 2.71 (95% CI: 1.54–4.79) times increased odds of goiter. Compared to nulliparous, women with parity of 1, 2 and 3 or more had 2.28 (95% CI: 1.01–5.16), 2.81 (95% CI: 1.17–6.74) and 4.41 (95% CI: 1.58–12.26) times higher risk of goiter. Compared to pregnant women aged 15–29 years, the prevalence of goiter was significantly higher among those aged 30–44 years (AOR of 2.32 (95% CI: 1.05–5.14)) (Table 4).

**Table 4:** Factors associated with goiter status among pregnant women in rural Ada districts, Oromia region, Ethiopia, November, 2014

Variables	Goiter	No goiter	OR (95% CI)	
			COR (95% CI)	AOR (95% CI)
Age (years)				
15–29	50	258	1^r^	1^r^
30–44	22	26	4.37 (2.29–8.31)^a^	2.32 (1.05–5.14)^a^
Maternal education				
Illiterate	44	116	2.27 (1.34–3.86)^a^	2.71 (1.54–4.79)^a^
Literate	28	168	1^r^	1^r^
Household wealth index				
Poorest	12	59	1^r^	1^r^
Poorer	17	54	1.54 (0.67–3.53)	1.92 (0.82–4.49)
Middle	11	61	0.88 (0.36–2.16)	1.11 (0.44–2.76)
Richer	15	56	1.31 (0.56–3.05)	1.77 (0.73–4.27)
Richest	17	54	1.54 (0.67–3.53)	1.99 (0.84–4.75)
Parity				
0	11	111	1^r^	1^r^
1	22	90	2.47 (1.14–5.36)^a^	2.28 (1.01–5.16)^a^
2	18	57	3.19 (1.41–7.20)^a^	2.81 (1.17–6.74)^a^
≥ 3	21	26	8.15 (3.50–18.98)^a^	4.41 (1.58–12.26)^a^
Gestational age				
First trimester	4	28	1^r^	1^r^
Second trimester	17	77	1.54 (0.48–4.99)	1.57 (0.45–5.50)
Third trimester	51	179	1.99 (0.67–5.95)	2.12 (0.65–6.87)
Kale consumption				
Three to six per week	18	81	1.07 (0.49–2.36)	1.12 (0.47–2.65)
Once or twice per week	41	140	1.41 (0.71–2.83)	1.41 (0.65–3.02)
Never	13	63	1^r^	1^r^
Cabbage consumption				
Three to six per week	12	68	0.78 (0.36–1.67)	0.95 (0.39–2.26)
Once or twice/week	37	114	1.43 (0.80–2.58)	1.29 (0.68–2.49)
Never	23	102	1^r^	1^r^
Sorghum consumption				
Three to six per week	14	51	1.12 (0.57–2.18)	0.98 (0.45–2.14)
Once or twice/week	6	20	1.22 (0.47–3.21)	0.91 (0.31–2.71)
Never	52	213	1^r^	1^r^
Awareness about iodized salt				
No	55	207	1^r^	1^r^
Yes	17	77	0.83 (0.45–1.51)	0.65 (0.31–1.36)
Awareness about goiter				
No	54	225	1^r^	1^r^
Yes	18	59	1.27 (0.69–2.32)	2.04 (0.96–4.35)
Time salt added during cooking				
In the beginning	13	64	1^r^	1^r^
Halfway through cooking	17	55	1.52 (0.67–3.41)	1.16 (0.48–2.83)
Towards the end	37	141	1.29 (0.64–2.59)	1.20 (0.56–2.57)
After cooking	5	24	1.02 (0.33–3.18)	0.89 (0.26–3.14)
Salt sun light exposure practice				
No	64	256	1^r^	1^r^
Yes	8	28	1.14 (0.49–2.62)	1.06 (0.41–2.77)
Iodine level of salt				
< 15 ppm(inadequate)	48	165	1^r^	1^r^
> 15 ppm(adequate)	23	115	0.68 (0.39–1.19)	0.75 (0.41–1.37)

1^r^ Reference group

^a^Significant association at 95% confidence level

## Discussion

This study provides evidence on the magnitude of iodine deficiency among a highly vulnerable population group with the use of both clinical and biochemical methods. The results from the present study showed widespread iodine deficiency among pregnant women in the study area.

The urinary iodine concentration represents recent iodine intake and is widely accepted as a good indicator of iodine status. According to the WHO/UNICEF/ICCIDD, a median UIC among pregnant women within the range of 150–249 μg/L is indicative of iodine sufficiency [2]. In the current study the median UIC was 85.7 μg/L which is lower than the recommended minimum median UIC for pregnant women. Moreover, 78% of the study subjects had UIC below the cutoff for iodine adequacy (150 μg/L) which suggests that even their unborn children are at risk of having IDDs.

Few studies have previously determined the iodine status of pregnant women in Ethiopia. According to Ersino and colleagues, in 2009 among 172 pregnant women in three rural kebeles in Sidama zone, prevalence of severe iodine deficiency (UIC < 20 μg/L) was 60% and the median UIC was 15 μg/L [12]. A study conducted in Haramaya, Eastern Ethiopia among 435 pregnant women, found a median UIC of 58.1 μg/L and 82.8% prevalence of subclinical iodine deficiency [13]. A hospital based cross-sectional study conducted in Jimma, South Western Ethiopia among 423 pregnant women reported, 88.9% prevalence of iodine deficiency and a median UIC of 48 μg/L [16]. These studies reported low median UIC and a high proportion of pregnant women with UIC less than 150 μg/L compared to the current study. Although the median UIC (85.7 μg/L) obtained in the current study was low, it shows there is a slight improvement over time. This may be due to the fact that our study was undertaken two years after the mandatory salt iodization program was launched in Ethiopia.

According to WHO/UNICEF/ICCIDD prevalence of goiter greater than 5% indicates a public health problem [2]. The prevalence of goiter in the current study (20%) greatly exceeded the cut-off where IDD is considered a public health problem and falls within the category of moderate iodine deficiency in the study area. However, prevalence of goiter in our population was lower than the reported national (36%) and regional prevalence of goiter rate (31.3%) reported in 2005 (10). Two other studies conducted in Sidama zone [12] and in West Gojjam [17] reported 48.5 and 30.1% goiter prevalence, respectively. The reduction in goiter in this study could be due to the implementation of iodized salt since the previous studies and geographical disparity in the country along with the probable environmental and dietary iodine deficiencies [18].

The higher rates of goiter found in the parous, as compared with the nulliparous in the current study are consistent with the understanding that repeated pregnancies could deplete women’s iodine status [19]. Previous studies conducted in Denmark [20] and Italy [21] also reported similar findings.

Prevalence of goiter was increased with the age of the women. Older women had the higher risk of goiter, compared to younger women. This is consistent with other studies that reported an increased thyroid enlargement with age in women in areas of severe or moderate to mild iodine deficiency [22, 23]. However, it has been suggested that age by itself has no effect on goiter but it could be due to the likelihood of repeated pregnancy with age [21].

Lack of education among pregnant women was associated with a twofold increased risk of iodine deficiency and goiter rate was found to be higher among illiterate than literate ones. A study in Denmark found lower occurrence of goiter among participants with higher education level [24] and a study in school children from Istanbul revealed a higher prevalence of iodine deficiency in students with less educated caretakers [25]. Possibly educated pregnant women are more likely to consume food with higher iodine level or to purchase iodized salt. A study in France in adults revealed an association between high educational level and lower risk of low iodine intake [26].

In order to alleviate IDD, universal salt iodization (USI) which is iodizing all salt for human and animal consumption is the best solution [2]. According to the Ethiopian Demographic and Health Survey (EDHS) only 15.4% of household salt samples in 2011 contained at least 15 ppm iodine [14]. In the present study 39.3% of the salt samples had iodine > 15 ppm, which shows some progress, however, a great deal of effort is still needed to reach the goal that at least 90% of the population should use adequately iodized salt (> 15 ppm iodine and < 40 ppm, at the household level) [2]. Two years after mandatory salt iodization intervention was launched in Ethiopia, more than 60% of the salt samples in the study area were below 15 ppm. Only 39.3% of households were consuming adequately iodized salt with 15 ppm or more iodine, and the iodine concentration of salt samples varied widely (0.9 to 191.2 ppm). The results of the present study are comparable with a national survey conducted in 2014 which reported the coverage of adequately iodized salt at the household was 42.7% nationally and 36.7% in the Oromia region where the study area is situated [27]. In view of the substantial under-iodization as well as the considerable variation in iodine concentration observed in household salt, the lack of knowledge about IDD and iodized salt among pregnant women in this study implies that the message about the extent of IDD including its effect on brain has not been conveyed successfully at the consumer level in the country.

To the best of our knowledge, this is the first study among pregnant women in Ethiopia after the introduction of mandatory iodization program describing iodine deficiency with the use of both clinical and biochemical methods, as well as estimating household salt iodine concentration using a quantitative method. However, the study has several limitations. A single urine sample was collected from each woman. Because there is substantial day to day variation of individual iodine intakes, UIC can only be used to assess the iodine status of a population, but not of the individuals [28]. Other nutrients including selenium, which may exacerbate the effects of iodine deficiency were not assessed in our study.

## Conclusions

Iodine deficiency was a public health problem among pregnant women in the study area. Goiter significantly associated with advanced parity, older age and illiteracy. Despite the progress in Ethiopia to prevent and control IDDs, the proportion of households consuming adequately iodized salt was lower than the internationally recommended values for control of IDDs. More importantly, there was a wide variation in iodine concentrations of salt samples. This indicates the need for further strengthening the existing salt iodization program in order to avail homogenously and adequately iodized salt. Also innovative ways to educate the people about the ill effects of IDDs and its preventive methods are urgently needed. Because pregnant women are one of the most vulnerable groups for IDD, it is necessary to find ways to provide iodine supplements as needed until USI is fully established.

## Abbreviations

EDHS: Ethiopian Demographic and Health Survey
EPHI: Ethiopian Public Health Institute
FAO: Food and Agricultural Organization
ICCIDD: International Council on Control of Iodine Deficiency Disorders
IDD: Iodine Deficiency Disorders
SAC: School Age Children
TGP: Total Goiter Prevalence
UIC: Urinary Iodine Concentration
USI: Universal Salt Iodization
WHO: World Health Organization

## References

[1] FAO/WHO. Vitamin and mineral requirements in human nutrition. 2nd ed. Geneva: World Health Organization; 2004.

[2] WHO/UNICEF/ICCIDD (2007). Assessment of iodine deficiency disorders and monitoring their elimination: A guide for program managers.

[3] Pearce EN, Andersson M, Zimmermann MB (2013). Global iodine nutrition:where do we stand in 2013?. Thyroid.

[4] Andersson M, Karumbunathan V, Zimmermann MB (2012). Global iodine status in 2011 and trends over the past decade. J Nutr.

[5] Glinoer D (2007). The importance of iodine nutrition during pregnancy. Public Health Nutr.

[6] Trumpff C, Schepper JD, Tafforeau J, Van Oyen H, Vanderfaeillie J, Vandevijvere S (2013). Mild iodine deficiency in pregnancy in Europe and its consequences for cognitive and psychomotor development of children: a review. J Trace Elem Med Biol.

[7] Yarrington C, Pearce E. Iodine and pregnancy. J Thyroid. 2011:1–8. 10.4061/2011/934104 PMC3134395 21765996

[8] Zimmermann MB, Jooste PL, Pandav CS (2008). Iodine deficiency disorders. Lancet.

[9] Zimmermann MB (2012). The effects of iodine deficiency in pregnancy and infancy. Paediatr Perinat Epidemiol.

[10] Abuye C, Berhane Y (2007). The goitre rate, its association with reproductive failur and the knowledge of iodine deficiency disorders (IDD) among women in Ethiopia: cross-section community based study. BMC Public Health.

[11] Adish A, Chuko T, Abay A, Assey V, Desta T (2013). Ethiopia: breaking through with a new iodized salt law. IDD Newsletter.

[12] Ersino G, Tadele H, Bogale A, Abuye C, Stoecker BJ (2013). Clinical assessment of goiter and low urinary iodine concentration depict presence of severe iodine deficiency in pregnant Ethiopian women: a cross-sectional study in rural Sidama, Southern Ethiopia. Ethiop Med J.

[13] Kedir H, Berhane Y, Worku A. Subclinical iodine deficiency among pregnant women in Haramaya district, eastern Ethiopia: a community-based study. J Nutr Metab. 2014;2014:1–8. 10.1155/2014/878926 PMC4124753 25132987

[14] Central Statistical Agency of Ethiopia, Measure DHS (2011). Ethiopia Demographic and Health Survey 2011.

[15] Gibson RS (2005). Principles of nutritional assessment.

[16] Zenebe N, Gobena T, Rajesh PN, Kassim M (2014). Determining the magnitude of iodine deficiency and its associated risk factors among pregnant women visiting Jimma University specialized hospital for antenatal care. World J Med Sc.

[17] Kebede A, Belay A, Ayana G, Tesfaye Y, Zilelew A, Abuye C (2014). Iodine deficiency disorders (IDD) in Burie and Womberma districts, west Gojjam, Ethiopia. AJFAND.

[18] Gebreegziabher T, Teyike N, Mulugeta A, Abebe Y, Hambidge KM, Stoecker BJ (2013). Lack of dietary sources of iodine and the prevalence of iodine deficiency in rural women from Sidama zone, Southern Ethiopia. AJFAND.

[19] Smyth PA, Hetherton MT, Smith DF, Radcliff M (1997). O’herlihy C. Maternal iodine status and thyroid volume during pregnancy. J Clin Endocrinol Metab.

[20] Knudsen N, Laurberg P, Perrild H, Bülow I, Ovesen L, Jørgensen T (2002). Parity is associated with increased thyroid volume solely among smokers in an area with moderate to mild iodine deficiency. Eur J Endocrinol.

[21] Rotondi MN, Amanto G, Biondi B, Mazziotti G, Del Buono A, Balzano S, Bellastella A, Glinoer D, Carella C (2000). Parity as a thyroid size determining factor in areas with moderate iodine deficiency. J Clin Endocrinol Metab.

[22] Adesunkanmi A, Makinde O (2003). Goiter prevalence in pregnant women attending antenatal clinic in a teaching hospital. J Obstet Gynecol.

[23] Knudsen N, Bülow I, Jørgensen T, Laurberg P, Ovesen L, Perrild H (2000). Goiter prevalence and thyroid abnormalities at ultrasonography: comparative epidemiological study in two regions with slightly different iodine status. Clin Endocrinol.

[24] Knudsen N, Bulow I, Laurberg P, Ovesen L, Perrild H, Jørgensen T (2003). Low socio economic status and familial occurrence of goitre are associated with a high prevalence of goiter. Eur J Epidemiol.

[25] Gür E, Ercan O, Can G, Akkus S, Guzeloz S, Ciftcili S, Arvas A, Iltera O (2003). Prevalence and risk factors of iodine deficiency among school children. Trop Pediatr J.

[26] Valeix P, Faure P, Peneau S, Estaquio C, Hercberg S, Bertrais S (2009). Lifestyle factors related to iodine intakes in French adults. Public Health Nutr.

[27] EPHI (Ethiopian Public Health Association) (2014). National salt iodization coverage towards prevention of iodine deficiency disorder in Ethiopia.

[28] Konig F, Andersson M, Hotz k AI, Zimmermann MB (2011). Ten repeat collections for urinary iodine from spot samples or 24-hour samples are needed to reliably estimate individual iodine status in women. J Nutr.

